# Ultrastructural Analysis of Differences in the Growth and Maturation of Staphylococcus pseudintermedius Biofilm on Biotic and Abiotic Surfaces

**DOI:** 10.1128/spectrum.03577-22

**Published:** 2023-02-13

**Authors:** Lopamudra Kher, Karen Kelley, Domenico Santoro

**Affiliations:** a Department of Small Animal Clinical Sciences, University of Florida, Gainesville, Florida, USA; b Interdisciplinary Center for Biotechnology Research (ICBR), Electron Microscopy Core Facility, University of Florida, Gainesville, Florida, USA; Weizmann Institute of Science

**Keywords:** biofilm, biotic surface, abiotic surface, canine skin explant, membrane filter, *ex vivo* model, *in vitro* model

## Abstract

Biofilms are extremely complex yet systematic microbial structures. Studies comparing the differences in their growth on living and nonliving surfaces by electron microscopy are limited. Therefore, the purpose of this study was to ultrastructurally investigate the differences in the growth and development of Staphylococcal biofilm on polycarbonate filters and canine skin explants. Using scanning and transmission electron microscopy (SEM and TEM), Staphylococcus pseudintermedius was incubated for 6, 12, 24, 48, and 72 h. It was observed that similar amounts of exopolymeric substance (EPS) were deposited on the biofilm on both surfaces, but the biofilm on the skin explants was primarily flat, whereas the biofilm on the membrane developed a multilayered plateaued look. Microcolony formation was only observed on the membrane filter during the early stages of biofilm development. On the membrane biofilms, EPS was observed to be deposited in a distinctive pattern. EPS deposition on the membrane surface was observed to peak before it declined, but on the explant, a constant increase was observed at all time points. Cell exposure to the environment on both the membrane filters and explants differed depending on the stage of biofilm formation. On both the membranes and the skin explants, there was a perceptible difference between the biofilm growth patterns and speeds. The results of this study suggest that data extrapolated from studies on biofilm bactericidal compounds performed on abiotic surfaces (such as polycarbonate filters) may not be entirely applicable to biofilm growing on biotic surfaces (e.g., skin) due to ultrastructural variations revealed in this study.

**IMPORTANCE** Biofilm has been recognized as an important source of antimicrobial resistance. These sessile microbial colonies tend to attach and grow on every surface, biotic and abiotic, and they account for approximately 80% of chronic and recurrent infections. Biofilms are not all alike; they have different structures and microbial compositions. This high variability allows for differences in the production of exopolymer substances, affecting antimicrobial penetration. No studies have been published that simultaneously compare the structure of biofilms grown on abiotic (*in vitro*) and biotic (*ex vivo*) surfaces. To identify treatment alternatives, it is essential to understand the differences between biofilms. The results of the study show how biofilm structures and compositions are dependent on the substrate on which they grow.

## INTRODUCTION

Antonie van Leeuwenhoek (1), a Dutch researcher, first identified microbial aggregates on scrapings from dental plaques in the 17th century; nevertheless, it was not until almost 2 centuries later that Characklis described these aggregates as exceedingly tenacious and resistant to standard disinfectants (2). Costerton et al. (3) coined the term “biofilm” to describe microbial aggregation in 1978. Biofilms were defined as microbial communities formed by sessile bacteria that irreversibly adhered to a biotic or abiotic surface using an additional polymeric substance (exopolymeric substance [EPS]) created by themselves (4). In nature, biofilms are composed of heterogeneous species (5); for example, a biofilm may contain multiple species of bacteria or be mixed with fungal species. The bacteria are distributed in highly organized microcolonies inside the biofilm, accounting for 15% to 20% of the biofilm mass, with EPS accounting for the remaining 75 to 80% of the biomass (4).

Biofilms are microbial structures that are extraordinarily complex and yet well organized. Bacterial biofilm is responsible for around 80% of chronic and recurring microbial illnesses in humans (6). This is because biofilm functions as a barrier, making the cells 10 to 1,000 times more resistant to antimicrobials than planktonic cells (6). Bacterial cells in a biofilm can also tolerate harsh environmental or host conditions, such as immunological reactions (7). All of these characteristics combined make biofilm-associated illnesses extremely difficult to cure and, as a result, a significant hazard to both animal and human health.

Biofilm formation is characterized by several essential chronological steps: (i) bacterial attachment (reversible to irreversible), (ii) microcolony formation, (iii) maturation, and finally, (iv) dispersal (2, 8–10). These steps were identified and studied on biofilms originally grown *in vitro* on nonliving/abiotic surfaces (such as plastic, microtiter plates, etc.). Although these *in vitro* investigations improved our understanding of the early processes involved in biofilm infections and structure, they did not mimic clinical biofilms. In fact, *in vitro* biofilm models have failed to explain the processes that allow biofilms to persist in chronic infections, making it difficult to extrapolate information to be applied to chronic illnesses (11, 12). To acquire more knowledge about clinical biofilm, *in vivo* models, in which biofilms are generated in or on a live animal (e.g., porcine—to understand the nature of wound healing) have been proposed as an alternative. These models resulted in biofilms that were similar to clinical biofilms and had characteristics distinct from the *in vitro* biofilm shape (*in vivo* biofilms lack the typical mushroom shape seen with *in vitro* biofilms) and size (*in vivo* biofilms [diameter, ~5 to 200 μm] are significantly smaller than *in vitro* biofilms [up to 1,200 μm]) (11). These differences are attributed not only to the presence of an active host immune response but also to the substrate and nutrient availability on a biotic surface, which is important for molding biofilm growth (11, 12). However, *in vivo* models, like *in vitro* models, have constraints such as cost (both experimentation and animal rearing), ethical considerations about animal usage, and difficulty in simulating long-term inflammatory and antibiotic responses, limiting their use (7, 11). Therefore, the next and last option for studying biofilms is the use of *ex vivo* models. In *ex vivo* models, biofilms are grown on a tissue of interest obtained from live animals (e.g., porcine—lung, skin tissue, etc.). The biofilms are grown in a controlled environment similar to that seen in *in vivo* models using *in vitro* methods and synthetic media (12). This type of model is a good blend of both *in vitro* and *in vivo* models. Nevertheless, it lacks the active host immune response seen in *in vivo* models. However, studies using such models are less expensive than *in vivo* studies.

*Ex vivo* models could be an excellent way to learn more about biofilm kinesis and develop more targeted therapy approaches than with *in vitro* models. However, there are currently no studies employing these two models to compare the growth and development of biofilm at the same time. Therefore, the objective of this study was to simultaneously characterize and compare the growth and development of biofilm over time on two different substrates (i.e., membrane filters [an abiotic *in vitro* model] and canine skin explants [a biotic *ex vivo* model]). The hypothesis to test was that there would be considerable differences in the biofilm structure, shape, EPS production, and biomass between the two surfaces.

## RESULTS

### Different forms of EPS.

**FIG 1:**
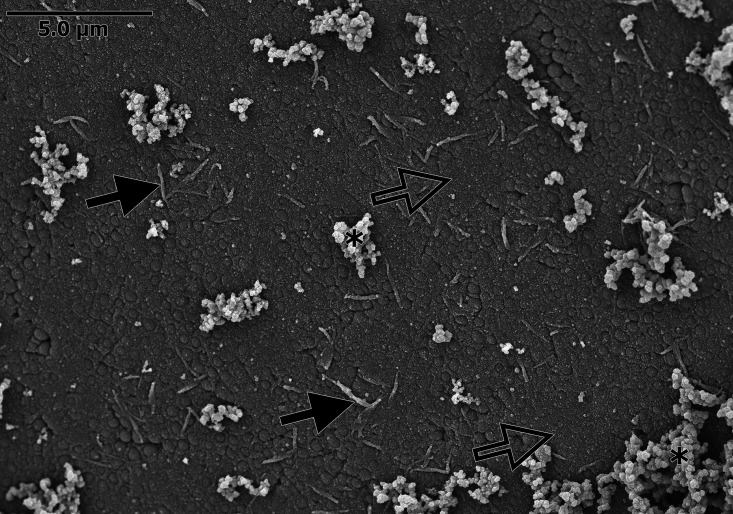
Scanning electron microscopy (SEM) image of Staphylococcus pseudintermedius biofilm grown on the surface of the membrane filter. Note that the bacterial cells are completely embedded in exopolymeric substance (EPS). Three forms of EPS are recognizable: spongy EPS (*), filamentous EPS (solid arrows), and compressed EPS (open arrows).

In this study, different forms of EPS (Fig. 1) were identified: (i) spongy EPS (sEPS; spongy with a rough surface, seen either associated with cells or separately), (ii) filamentous EPS (fEPS; filamentous cords connecting cells), and (iii) compressed EPS (cEPS; a fine layer of uniform-appearing EPS, acting as cement between cells). cEPS was formed by coalescing and compression of sEPS, which was noticeable at increasing magnification.

### Scanning electron microscopy images of biofilm grown on a polycarbonate membrane filter.

**FIG 2:**
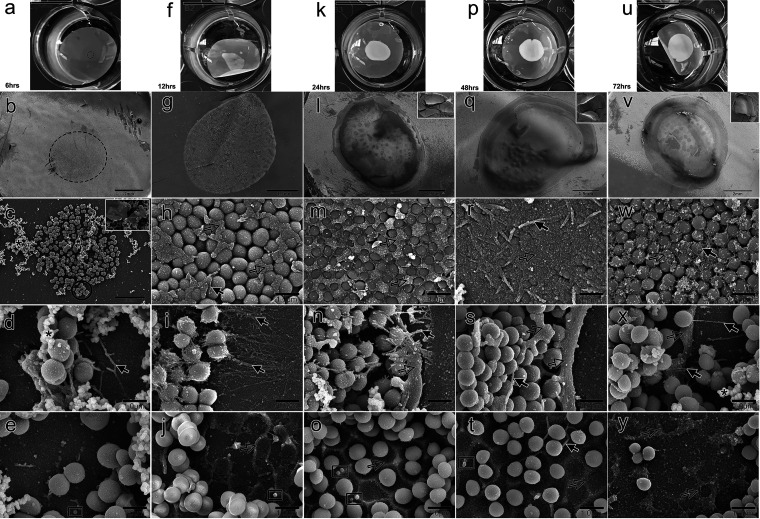
Using scanning electron microscopy (SEM), transverse views were obtained of a biofilm formed by Staphylococcus pseudintermedius over 72 h. Images show the biofilm growth pattern at 6 h (a to e), 12 h (f to j), 24 h (k to o), 48 h (p to t), and 72 h (q to y). The upper row (a, f, k, p, and u) shows the macroscopic appearance of the biofilm over 72 h. (b, g, l, q, and v) Low magnification view of the biofilm growth over time from 6 to 72 h. (c) Microcolonies (black circle) were observed either exposed to the environment or covered by exopolymeric substance (EPS). (h, m, r, and w) The surface of the multilayer biofilm was gradually coated by compressed EPS (h, m, and r), but after 72 h, the surface was once again exposed due to EPS depletion (w). (d, i, n, s, and x) At the perimeter, the thickness of the biofilm was reduced to a few cell layers (tapered) and was held in place by filamentous EPS (d). Over time, the density of the filamentous EPS grew (i and n), and it was gradually replaced by compressed EPS (n and s). At 72 h, the density of the compressed EPS decreased, and daughter cells were observed to be spanning the perimeter to create new colonies (x). (e, j, o, t, and y) Cells were gradually seen etched in the EPS as they accumulated (e, j, o), creating a honeycomb structure (o). This pattern degraded between 48 and 72 h (t and y). Outer membrane vesicles (black boxes) were seen released in the EPS (e, j, o, and t). *, spongy EPS (sEPS); solid arrows, filamentous EPS (fEPS); open arrows, compressed EPS (cEPS); dotted circles indicate the area of inoculation.

**(i) At 6 h.** Bacterial growth was not visibly apparent after 6 h (Fig. 2a); however, at lower magnification, the margins of the inoculation area were evident (Fig. 2b). The surface of the membrane filter was covered with cEPS, and cocci were seen scattered in clusters.

Microcolonies with a thickness of two to three cell layers formed clusters with wide empty spaces between them (Fig. 2c). sEPS was observed at the edges or covering the cells (Fig. 2c and d). The cells were held in place by the fEPS, which was observed to emerge from the cEPS (Fig. 2d). Strands of fEPS were also seen connecting cells farther away from it. A few outer membrane vesicles (OMVs) were seen dispersed on the surface of cEPS at the bottom (Fig. 2e).

**(ii) At 12 h.** The bacterial multiplication was visible after 12 h (Fig. 2f). At reduced magnification, the biofilm’s microscopic surface resembled a barren landscape (Fig. 2g). Higher magnification revealed the loss of the microcolonies and the replacement of the large empty gaps by daughter cells (Fig. 2h). The spaces between neighboring cells on the topmost layer were filled with cEPS (Fig. 2h). The biofilm was seen to taper off at the edge to just a few cell layers. A denser network of fEPS strands was seen holding those cells in place (Fig. 2i). As the cEPS accumulated, it was observed at the bottom forming a honeycomb-like pattern where the cells once resided (Fig. 2j).

### Transverse view.

As the biofilm continued to grow, the top layer lifted off during processing, exposing the cells and structures beneath.

**(i) At 24 h.** The bacteria continued to multiply (Fig. 2k and l). In addition, cEPS started filling all the open areas between the cells and partially enveloping the cells (Fig. 2m). The biofilm cells at the perimeter were surrounded by a thick rim of cEPS, from which fEPS was visible extending and holding the cells in place (Fig. 2n). The cells could be seen engraved inside the cEPS at the basal layer, making the honeycomb pattern and OMVs visible (Fig. 2o).

**(ii) At 48 h.** As the biofilm grew, it continued to spread horizontally (Fig. 2p and q). The cEPS nearly engulfed the exposed cells, and thick strands of fEPS were visible emerging from it, linking the cells together (Fig. 2r). The fEPS observed at 24 h was replaced by cEPS at the perimeter (Fig. 2s). The cEPS thickness at the basal layer was seen to be lesser than it was after 24 h (Fig. 2t).

**(iii) At 72 h.** Even though the biofilm kept growing (Fig. 2u and v), the exposed cells were no longer protected by cEPS and could now be observed to be interconnected by a thin network of fEPS. sEPS was observed either depositing on fEPS or the surface of a few cells (Fig. 2w). Cells were seen crossing over the side, and the band of cEPS at the perimeter was diminished. These cells had sEPS on their surface and were connected by fEPS (Fig. 2x). The cEPS had decreased at the base, but it was still possible to see evidence of its previous presence (Fig. 2y).

### Pattern of EPS position (longitudinal view).

The complete thickness of the biofilm exhibited an odd pattern of EPS deposition as it developed.

**FIG 3:**
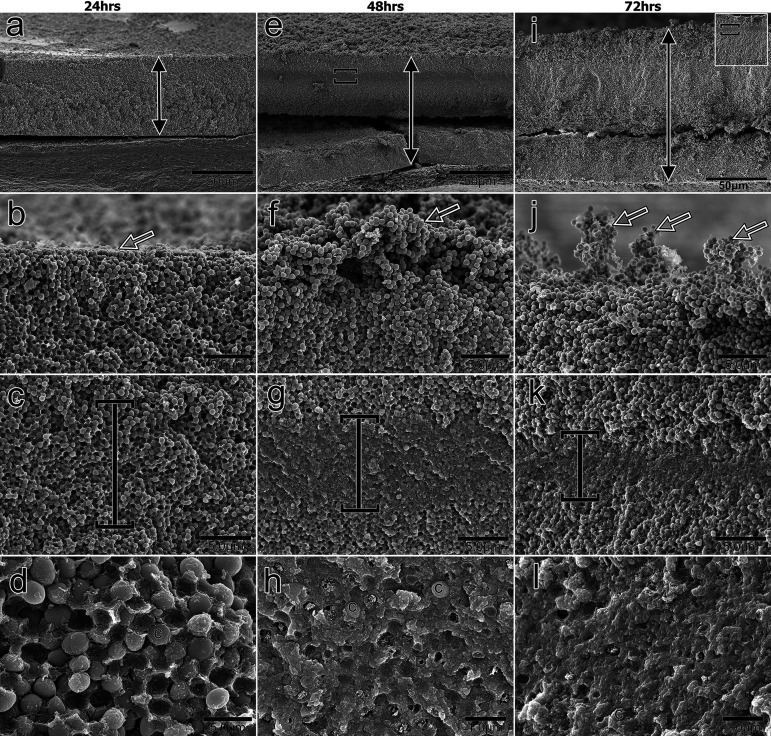
Using scanning electron microscopy (SEM), longitudinal views were obtained of a biofilm formed by Staphylococcus pseudintermedius over 72 h. An alternate band of exopolymeric substance (EPS) deposition was seen from 24 to 72 h. Over time, the biofilm’s thickness grew (a, e, i), indicated by pointed black arrows. The biofilm’s surface started flat (b) and progressively rose over time (f), with bacterial cells forming loosely connected clusters (j) ready to be dispersed in the environment. A well-defined zone of EPS (vertical square brackets) was seen (c, g, and k). This zone decreased in thickness, while increasing in density over time (c, g, and k), making the cells trapped inside less visible (d, h, and l). Bacterial cells are labeled with a “c”; the open white box in panel i provides a low magnification view of the biofilm.

**(i) At 24 h.** The biofilm was divided into two halves, and the surface was flat (Fig. 3a and b). Although it looked like the cells were covered with EPS throughout the thickness, this density of EPS deposition was visible inside the biofilm in alternate layers (Fig. 3c and d). While the cells in the central area had a dense coating of EPS (Fig. 3d), the cells immediately above and below had a thin coating of EPS.

**(ii) At 48 h.** Although the biofilm was divided into two sections, an area of heavily deposited EPS could be seen in the top part (Fig. 3e). Higher magnification revealed a rich layer of cEPS coating the cells in this area (Fig. 3g and h). In contrast to the biofilm’s flat surface at 24 h, the cells at the top looked to be slightly elevated, forming a gap beneath them (Fig. 3f).

**(iii) At 72 h.** Once again, two horizontal sections of the biofilm were visible (Fig. 3i). The pattern of EPS deposition resembled that observed at 48 h (Fig. 3i, k, and l). The cells on the surface were now visible as loose flocs that were loosely adhered to the biofilm with the aid of sEPS, as opposed to the cells that had previously been seen as slightly lifted (Fig. 3j).

### TEM images of biofilm growth on the surface of the polycarbonate membrane filter.

**FIG 4:**
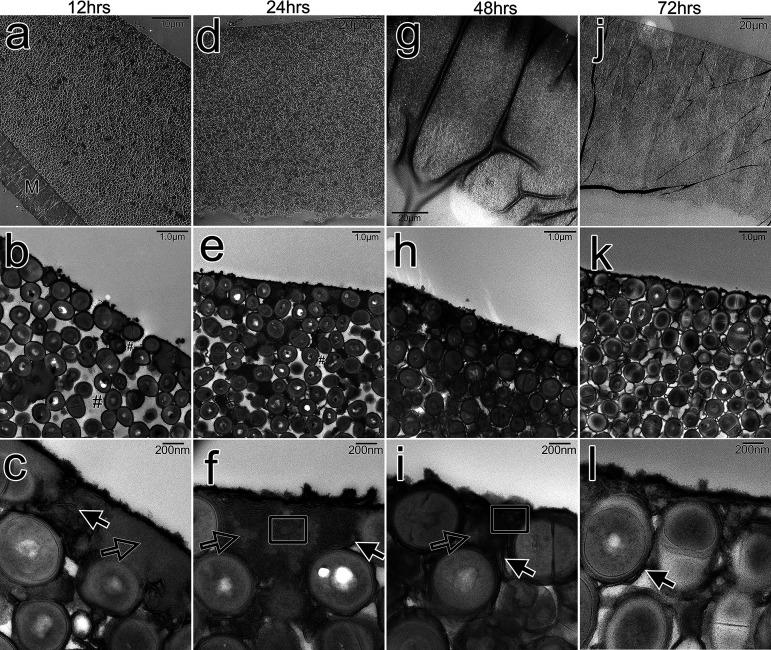
Using transmission electron microscopy (TEM), Staphylococcus pseudintermedius biofilm was examined from 12 to 72 h. The biofilm initially adhered to the membrane surface (a), but as it thickened, it was observed to detach from it (d, g, and j). Over time, the cEPS appeared as an electron-dense gel-like substance that filled the voids between cells (b, e, and h). At 72 h, as the biofilm matured, the layer of EPS decreased in thickness (k). The majority of filamentous EPS was shown connecting cells, while extracellular vesicles were observed trapped in the compressed and filamentous EPS network (c, f, i, and l). M, polycarbonate membrane surface; #, voids; open boxes, outer membrane vesicles; solid arrows, filamentous EPS; open arrows, compressed EPS.

TEM images were not taken at 6 h, since the biofilm was thin and just 2 to 3 cell layers thick. The biofilm thickened and was multilayered at 12 h (Fig. 4a). The cells were observed to be actively multiplying and to be electron dense (Fig. 4b). sEPS was seen adhered to the surface of the cells (Fig. 4a). In the top layer of the biofilm and some locations inside the biofilm, it was noticed that cEPS were filling the spaces between the cells (Fig. 4a), but the remaining spaces remained empty (Fig. 4b). Strands of fEPS were occasionally seen in between cEPS in the topmost layer (Fig. 4c).

At 24 h, the biofilm detached from the membrane surface as its thickness increased (Fig. 4d). The cells could be seen growing in number, and cEPS had taken up a sizable amount of space between them (Fig. 4d and e). Nevertheless, certain areas were still without cEPS (Fig. 4e). OMVs were observed to be entrapped within the cEPS (Fig. 4f).

The biofilm was once considerably electron dense (Fig. 4g). At 48 h, most of the vacant spaces were occupied by the cEPS (Fig. 4g and h). Higher magnification revealed filamentous strands of fEPS linking the cells together inside cEPS (Fig. 4h and i), along with a few OMVs (Fig. 4i).

Even though the cells were shown to be multiplying at 72 h (Fig. 4j), the biofilm as a whole had less electron density. Although fEPS was still visible in the connecting cells (Fig. 4l), the cEPS had significantly decreased (Fig. 4k).

### SEM images of biofilm grown on a canine skin explant.

Two distinct fixative methods were utilized: the standard aldehyde/osmium tetroxide method (with paraformaldehyde [PF] and glutaraldehyde [GA]) (PF-GA-OsO_4_) and the ruthenium red (RR)-lysine method (PF-GA-RR-lysine). PF-GA-RR-lysine was used to preserve the EPS, reducing detail loss during scanning electron microscopy (SEM) processing. In contrast, the standard PF-GA-OsO_4_ fixative did not completely preserve the EPS, and some detail loss was observed. The standard method aided in the observation of the microbial structures below the EPS layers.

**FIG 5:**
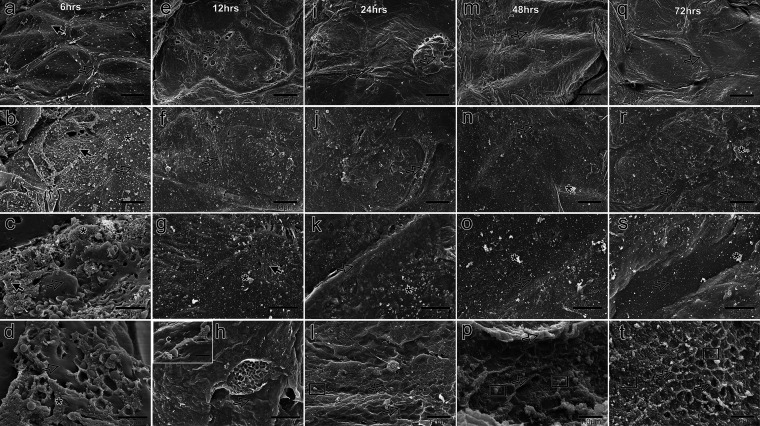
Using scanning electron microscopy (SEM), Staphylococcus pseudintermedius biofilm was examined from 6 to 72 h on the surface of a canine skin explant. The corneocyte surface was hidden when the biofilm expanded due to the released EPS. Osmium tetroxide (a, e, i, m, and q) and ruthenium red (b, f, j, n, and r) were used as fixatives. Increased cEPS deposition was seen at the corneocytic junction over time (c, g, k, o, and s). A honeycomb pattern was apparent at the bottom of these corneocytic junctions, where cells were observed to be trapped (d, h, l, p, and t). At 6 h (d) and 72 h (t), this pattern was visible, although it was smudged at other times (h, l, and p). *, spongy EPS (sEPS); solid arrows, filamentous EPS (fEPS); open arrows, compressed EPS (cEPS); black c, bacterial cell; white c, bacterial cell beneath EPS; open boxes, outer membrane vesicles.

At 6 h, a thin layer of a combination of cEPS and fEPS had formed on the surface of the corneocytes (Fig. 5a and b). In contrast to the membrane filter, the corneocytes showed very few cells on their surface, unlike the microcolony growth that was observed on the membrane filter. On the surface of the EPS, there were a few pores and blisters (Fig. 5b and c). sEPS could be seen throughout the surface (Fig. 5a and b). The EPS seemed more concentrated between corneocytes (intercorneocyte spaces) (Fig. 5a, c, and d). A substantial amount of cEPS was deposited between these spaces, and a microcanalicular system (honeycomb pattern) of EPS was visible at the base that resembled that seen on the membrane filters at 12 h (Fig. 5d). There were only a few cocci embedded in this EPS compared to that on the membrane filter (Fig. 5d).

By 12 h, the amount of EPS covering the explant surface had dramatically increased, blocking the view of corneocytes beneath it (Fig. 5e and f). Additionally, there were more visible surface blisters (Fig. 5e). Cocci were scarcely, if at all, visible on the surface (Fig. 5f). The amount of EPS filling the crevices between the corneocytes increased (Fig. 5g), making the honeycomb pattern less distinct than it was after 6 h (Fig. 5h). But cells were seen trapped underneath or on the surface of cEPS (Fig. 5h).

It became increasingly challenging to see corneocytes at 24 h, as the cEPS that had formed on the surface of the explants became denser and thicker (Fig. 5i and j). At this point, even fewer cells were visible on the surface (Fig. 5j). The spaces between the corneocytes were almost filled with EPS, which resembled cement (Fig. 5k). Though almost completely lost, some components of the microcanalicular system with OMVs could be seen (Fig. 5l).

By 48 h, the EPS covering the surface of the explant was even denser than it had been earlier (Fig. 5m and n). At this point, no cocci were visible on the surface. On the surface, there were clusters of sEPS. More EPS was deposited into the spaces between the corneocytes than in the previous 24 h, and this process began to gradually mimic the flow of a river between two pieces of land (Fig. 5o). The honeycomb pattern was once again visible at the base of the intercorneocytic gaps, although less clearly than after 12 h; OMVs were seen dispersed in the gaps, and no cells were observed wedged into them (Fig. 5p).

By 72 h, the EPS had continued to grow in volume, covered the explant completely, and filled in the crevices between the corneocytes, suggesting a river flowing across land (Fig. 5q, r, and s). Despite being present between the corneocytic spaces, the honeycomb pattern once again did not indicate the presence of any cells wedged in between the spaces, but a few OMVs were seen (Fig. 5t).

## DISCUSSION

This was the first investigation comparing biofilm formation using an *in vitro* (abiotic surface) and an *ex vivo* (biotic surface) model. It was observed that the biofilm formation on both of these surfaces was unique and had a distinct growth pattern (Table 1). Three distinct forms of EPS subtypes were identified: sEPS, fEPS, and cEPS. The sEPS was seen in the form of globular particles with a rough surface initially on the bacterial cell (13) that presumably was gradually released into the environment. This has shared characteristics with the EPS produced by Scardovia wiggsiae biofilm (14). The globules gradually merged to form the cEPS, which in the TEM images was seen as a gel-like substance and in the SEM images as a coarse cement. The cocci were seen initially dispersed on a layer of cEPS. The cEPS was seen engulfing the cells as the biofilm expanded. As the name implies, the fEPS developed a dense network of filaments connecting cells; environmental DNA (eDNA) has been demonstrated to localize in this filamentous structure (15). Additionally, fEPS seemed to act as a support structure on which sEPS accumulated before eventually coalescing into cEPS.

**TABLE 1:** Comparison of various biofilm growth characteristics over time on membrane filters (abiotic) and canine skin explants (biotic)^*a*^

Characteristic(s)	Description of appearance at:									
	6 h		12 h		24 h		48 h		72 h	
	Membrane	Explant	Membrane	Explant	Membrane	Explant	Membrane	Explant	Membrane	Explant
Microcolonies	Present	Absent	Absent	Absent	Absent, multilayered	Absent	Absent, multilayered	Absent	Seen forming outside the original biofilm	Absent
Multilayered biofilm	Absent	Absent	Present	Absent	Present	Absent	Present	Absent	Present	Absent
Cell exposure	Exposed	Very few exposed	Exposed	Very few exposed	Partially exposed	Barely exposed	Majority protected	Barely exposed	Partially exposed	None
EVs	Present	Present	Present	Present	Present	Present	Present	Absent	Absent	Absent
Microchannels/pores	Wide, present	Few	Narrow, present	Many	Barely seen	Many	Absent	Few	Void spaces	Absent
Shape		Flat	Plateau-like	Flat	Plateau-like	Flat	Plateau-like	Flat	Plateau-like, with loose floc of cells on the surface	Flat
Honeycomb pattern at the base	Absent	Present	At early stage of development	Present (less evident)	Present	Present	Present	Present	Absent	Present
Pattern of EPS deposition					Alternate, less dense; more fEPS than cEPS		Alternate, dense; mainly cEPS		Alternate, dense; mainly cEPS	
Surface		Flat		Flat	Flat	Flat	Bumpy, cells slightly raised	Flat	Clusters of cells loosely adhered	Flat

a Empty cells indicate that no pattern was discernible. EVs, extracellular vesicles; EPS, exopolymer substance; fEPS, filamentous EPS; cEPS, compressed EPS; sEPS, spongy EPS (seen scattered throughout at all time points on both surfaces, with no specific pattern).

The SEM was used at all time points, because it was challenging to choose sites for TEM due to the sparse scattering of bacterial cells visible across the surface (16).

While the explant did not exhibit characteristic biofilm shapes and had a level surface throughout, the biofilm on the membrane surface mainly had a plateau-like appearance but eventually revealed the presence of loosely linked clusters of cells that were prepared to be dispersed into the environment.

Although the EPS produced by the biofilm grown on the membrane filters and explants was comparable, the biofilm on the membrane filters had a multilayered thickness, whereas the biofilm on the explants did not. In contrast to the explants, which did not show microcolony formations at any time point, microcolonies (2 to 3 cell layers) were visible on the membrane filters, but only at the early stage of biofilm growth (6 h). Microcolony formation from the dispersed daughter cells of the biofilm was observed later in the developmental stage (72 h). Wide empty areas were visible between the microcolonies in the early stages of growth. These large empty gaps may act as microchannels that facilitate the passage of toxins and nutrients throughout the biofilm (17), although when the biofilm expanded, cEPS replaced these spaces in the channels. On the explant surface, pores (or blisters) were visible on the biofilm’s EPS; these pores may serve a similar purpose to the microchannels on a membrane filter. The number of pores increased over time (up to 24 h), and they gradually disappeared by 72 h. As the biofilm on the membrane surface continued to grow and mature, the number of exposed cells significantly decreased until 48 h. However, the EPS coating was seen to be diminished again at 72 h, exposing the cells. A distinctive EPS deposition pattern was observed both transversely and longitudinally on membrane filters. A microcanalicular structure resembling a honeycomb, similar to that formed by Streptococcus mutans (18), was seen transversely as the biofilm expanded, but with time, this pattern decreased. On the contrary, on the explants, a small number of cells were initially visible exposed on the surface and embedded in the honeycomb pattern, but as the biofilm expanded, all the cells were covered in EPS, leaving no cells exposed. Although EPS was visible longitudinally in all layers of the biofilm on the membranes, some areas had heavier and thicker EPS, enveloping the cells.

Staphylococcus quorum sensing (QS) could account for the difference in EPS density seen over time on the explant and membrane surfaces. The Staphylococcus aureus Agr QS system has the opposite impact on the growth of biofilms. Phenol-soluble modulins (PSMs) and matrix-degrading enzymes, which are highly involved in biofilm architecture and staphylococcal cell separation from the mature biofilm, are upregulated as a result of its activation (19). On the membranes, an increase in the number of cells possibly led to the activation of QS, causing the release of enzymes responsible for degrading EPS. In contrast, on the explants, there was no significant increase in the number of cells, potentially causing the downregulation of Agr QS, which in turn may have caused EPS to deposit more heavily on the corneocytes and intercorneocytic gaps and led to the formation of biofilm (19).

OMVs were detected scattered throughout the biofilm in the EPS, although they were not visible after 72 h. OMVs were observed in the EPS and on the cell surfaces as early as 6 h. OMVs are lipid-coated vesicles that cells secrete into the extracellular environment (20, 21). They were once thought to be a way for the biofilm to eliminate extracellular components but are now recognized as a new form of intercellular communication (21). Thus, it was not unusual to discover them in biofilm.

According to the findings of this study, it would not be prudent to extrapolate data from *in vitro* models for the treatment of clinical biofilms. Rather, data from *ex vivo* and *in vivo* models would be more trustworthy for this purpose.

One of the major limitations of this study was the inability to perform a TEM analysis on the biofilm that grew on the canine skin explants. This made it difficult to identify the different stages of biofilm formation. The different sterilizing techniques used for the two substrates was another drawback of this investigation. While the polycarbonate filter paper was sterilized in an autoclave, the explants were sterilized using chlorine gas. The different sterilization methods were dictated by the necessary use of poly-l-lysine coating on the membranes. This coating was necessary to increase bacterial adhesion and avoid the loss of the biofilm during the microscopic analysis; bleach would have altered its integrity. On the opposite, the skin samples could not be autoclaved to preserve the tissue integrity. Therefore, it is possible that the different biofilm growth rates observed on the two surfaces may be partially linked to the sterilizing procedure.

### Conclusion.

The findings of this study indicate that there are differences in the growth of biofilm on the surface of filters (abiotic surface) and canine skin explants (biotic surface), highlighting similarities and differences between *in vitro* versus *ex vivo* models. Both models showed the presence of three types of EPS (sEPS, fEPS, and cEPS) and the presence of voids/spaces or pores that were eventually replaced with EPS, indicating the presence of microchannels. However, many structural differences were also observed between the two models. The primary difference was that bacteria proliferated more quickly and were exposed to the surface on the membrane filters. On the skin explants, however, the cells proliferated more slowly and were primarily EPS coated. This is an important difference with significant implications for the type of therapy and therapeutic agents used in clinical practice. In conclusion, this study supports the use of the *ex vivo* model on biotic surfaces as more realistic and closer to the bacterial biofilm seen in clinical practice, whereas the *in vitro* model is better suited for studying biofilms in the environment or on abiotic surfaces. *Ex vivo* models should be given preference over *in vitro* models when testing antimicrobials designed to act on biofilm growing on biotic surfaces.

## MATERIALS AND METHODS

### Bacterial isolate.

Staphylococcus pseudintermedius ATCC 49444 (methicillin-susceptible Staphylococcus pseudintermedius [MSSP]) was used for this study. Bacteria were grown overnight on Columbia blood agar plates (Hardy Diagnostics, CA, USA) at 37°C in an incubator. Isolated colonies were suspended in sterile double-distilled water to achieve an optical density equal to a 0.5 McFarland standard using the Sensititre nephelometer (Thermo Fisher Scientific, Waltham, MA, USA).

The bacterial suspension was further diluted (1:100) in sterile HyClone Dulbecco’s phosphate-buffered saline (DPBS)/modified (GE Healthcare Life Sciences) to obtain a final concentration of ~1 × 10^6^ CFU/mL to be used on both surfaces.

### Biofilm growth on polycarbonate transmembrane filters (*in vitro* abiotic model).

To facilitate bacterial adherence during microscopy processing, Isopore Millipore polycarbonate membrane filters (diameter, 13 mm; pore size, 0.2 μm; Merck KGaA, Darmstadt, Germany) were submerged in a 0.01% (vol/vol) poly-l-lysine solution for 5 min and air-dried. Once the filters were dry, they were autoclaved at 121°C at 15 lb for 15 min. Poststerilization, they were carefully placed on soft tryptic soy agar. Then, 5 μL (~1 × 10^6^ CFU/mL) of the bacterial suspension was placed on the filters, which were incubated at 37°C and saturated humidity for 72 h to reach a mature biofilm state, as previously reported (22). The filters were sequentially removed after 6, 12, 24, 48, and 72 h and placed into 4% paraformaldehyde and 1% glutaraldehyde in 0.1 M cacodylate buffer, pH 7.24, a fixative for electron microscopy analysis. This experiment was run in triplicate.

### Biofilm growth on canine skin explants (*ex vivo* biotic model).

All experiments were approved by the Institutional Animal Care and Use Committee at the University of Florida (IACUC protocol number 201810132). All the skin explants were freshly harvested from the lateral abdominal region of dogs euthanized for medical reasons unrelated to this study, using 8-mm sterile disposable biopsy punches. The samples were washed in tap water and stored at −80°C until further use, as previously described (13). The methodology for this experiment was adapted from a previously validated and optimized methodology for S. pseudintermedius culture using a canine skin model (22). Briefly, the frozen skin explants were slowly brought to room temperature, and using a sterile size 11 stainless steel surgical blade (Integra Miltex, PA, USA), abrasions were created on the epidermis to mimic a wound to facilitate biofilm adhesion. The samples were then washed with tap water, transferred into a 50-mL sterile centrifuge tube (Corning Science Mexico, S.A.) containing 30 mL of 70% ethanol (30 explants/tube; ~1 mL/explant), and kept refrigerated at 4°C overnight. The following day, the tube was vortexed for a few seconds; the explants were then transferred into another sterile 50-mL centrifuge tube with 30 mL of fresh 70% ethanol and refrigerated overnight. On the third day, the explants were carefully placed on sterile gauze and sterilized using a mixture of chlorine and acetic acid (1:2 dilution) in a sealed gas chamber (SP Bel-Art Space Saver, H-B Instrument) for 45 min. Poststerilization, the explants were transferred into a centrifuge tube (using 2-μm nylon syringe filters by Thermo Scientific) containing a sterile 10% bleach/PBS/Tween solution (100 mL PBS, 100 mL bleach, 1 mL of 5% Tween 80, 1,000 mL sterile deionized water). The explants were then washed twice in sterile deionized water (5 min each time) before being carefully placed on soft tryptic soy agar. Then, 5 μL (~1 × 10^6^ CFU/mL) of bacterial suspension was placed on the explants, which were incubated at 37°C and saturated humidity for 72 h to achieve mature biofilms (22). Similarly to the membrane filter experiment, the skin explants were removed and placed into 4% paraformaldehyde and 1% glutaraldehyde in 0.1 M cacodylate buffer, pH 7.2, after 6, 12, 24, 48, and 72 h of incubation for electron microscopy analysis. This experiment was run in triplicate.

### Electron microscopy.

After exposure to bacteria, the aldehyde-fixed biofilm filters and skin explants were processed for ultrastructural examination by electron microscopy using two sample processing techniques: the standard aldehyde/osmium tetroxide fixation method and the ruthenium red-lysine method (23). All processes were carried out at room temperature. The washing and dehydration steps were conducted with the aid of a Pelco BioWave laboratory microwave (Ted Pella, Redding, CA, USA).

**(i) Standard aldehyde/osmium tetroxide method.** After fixation with 4% paraformaldehyde and 2.5% glutaraldehyde in cacodylate buffer, the filters and explants were washed with 0.1 M cacodylate buffer, postfixed with 2% buffered osmium tetroxide, water-washed, and dehydrated in a graded ethanol series of 25% through 100% with increasing concentrations of 5% to 10%.

**(ii) Ruthenium red-lysine method.**This method consisted of 1% glutaraldehyde and 500 ppm ruthenium red in a buffer composed of 0.1 M cacodylate and 50 mM lysine, pH 7.2. The samples were washed with buffer thrice, omitting the ruthenium red and lysine. The samples were postfixed with cacodylate-lysine–buffered 1% osmium tetroxide and 500 ppm ruthenium red and then buffer-washed, water-washed, and dehydrated in a graded ethanol series.

### Scanning electron microscopy.

After the membranes and explants were ethanol dehydrated, the samples were critical point dried, including overnight stasis mode (Autosamdri-815; Tousimis, Rockville, MD, USA). The dried filters and explants were mounted on carbon adhesive tabs on aluminum specimen mounts and gold-palladium sputter coated. The specimens were examined by secondary electrons (SE) on a field emission SEM (SU-5000; Hitachi High Technologies America, Schaumburg, IL, USA).

### Transmission electron microscopy.

To avoid loss of the biofilm colony through subsequent processing, the fixed biofilm membrane filters were sodium cacodylate buffer washed and encapsulated with low-temperature gelling agarose, type VII-A (Sigma-Aldrich, Saint Louis, MO, USA), followed by osmium tetroxide or ruthenium red, as described above (24). Postethanol dehydration was followed by anhydrous acetone exchanges and infiltration and embedding with Araldite 502/Embed 812 epoxy resin containing Z6040 embedding primer (Electron Microscopy Sciences, Hatfield, PA, USA). The skin explants were processed as described above; during the post-osmication washes, 1-mm^3^ pieces were excised, dehydrated, resin infiltrated, and embedded. The resin-infiltrated samples were cured for 72 h at 60°C, and semithick sections (500 nm) were then stained with toluidine blue. Ultrathin sections were collected on a carbon-coated Formvar 100 mesh grid (EMS, Hatfield, PA, USA).
